# Suitability of individual and bulk milk samples to investigate the humoral immune response to lumpy skin disease vaccination by ELISA

**DOI:** 10.1186/s12985-020-01298-x

**Published:** 2020-03-05

**Authors:** Milovan Milovanović, Vesna Milićević, Sonja Radojičić, Miroslav Valčić, Bernd Hoffmann, Klaas Dietze

**Affiliations:** 1 Department of Infectious Diseases of Animals and Diseases of Bees, University of Belgrade, Faculty of Veterinary Medicine, Blvd. Oslobodjenja 18, Belgrade, 11000 Serbia; 2 Virology Department, Institute of Veterinary Medicine of Serbia, Vojvode Toze 14, Belgrade, 11000 Serbia; 3 Friedrich-Loeffler-Institut, Südufer 10, D-17493 Greifswald-Insel Riems, Germany

**Keywords:** Lumpy skin disease, ELISA, Milk, Herd screening, Surveillance, Serology, Non-invasive

## Abstract

**Background:**

The detection of antibodies against capripoxvirus has become easier with a commercially available ELISA validated for serum and plasma. In order to explore its suitability for immunological investigations on alternative samples, this study targeted milk as sample matrix available through non-invasive sampling.

**Methods:**

Samples for this study were collected from dairy cows vaccinated against LSD in an area without reported LSD virus circulation. Paired serum and milk (individual and bulk) samples were tested by ELISA without and with modifications of the sample incubation time for the milk samples. For the evaluation of the test specificity, 352 milk samples from a milk repository in Germany were used as negative control. Receiver operating characteristic analysis was performed for determination of the Youden index and determination of the most suitable cut-off value for maximum specificity.

**Results:**

From 154 analyzed serum samples from Serbia, 75 were detected as positive in the ELISA. Sensitivity and specificity of the ELISA test for milk samples reached values of 88 to 91% using Youden criteria. A cut-off of 10 was determined aiming for maximum specificity. This cut-off value was used for further analysis. Using the protocol for serum, out of 154 milk samples, 38 were detected as positive, number of positive detected milk samples increase up to 48 with modified protocol. Milk samples from Germany reacted negative, except two samples that had borderline results using modified protocol. Significant statistical difference (*p* < 0.05) was observed between two incubation protocols. The detection of LSD-specific antibodies from bulk milk samples (pools of 2–10 individuals) came along with a reduced sensitivity over the sample of individual animals.

**Conclusions:**

Results show that the detection of capripoxvirus specific antibodies in milk samples using the commercially available ELISA from IDvet is feasible and can represent a helpful tool for LSDV monitoring programs.

## Introduction

Lumpy skin disease (LSD) is a vector-borne disease of cattle caused by lumpy skin disease virus (LSDV) which belongs to genus *Capripoxvirus* [1, 2]. Clinically, the disease can manifest in a wide spectrum ranging from severe acute to sub-acute and in-apparent forms. Typical signs observed are fever, enlargement of lymph nodes, nasal discharge, and firm skin nodules [3]. The control of LSD is mainly based on mass vaccination of the susceptible cattle population with live attenuated capripoxvirus vaccine [4, 5].

Recent studies suggest that LSDV vaccination stimulates equally cell mediated and humoral immunity [6–8]. The humoral immune response is of paramount practical importance to obtain information on the immune status of animals post infection or vaccination. The detection of antibodies against LSDV is possible starting one to 2 weeks post vaccination, increases gradually until 35 days to 12 weeks post vaccination, and is described to last until 40 weeks post vaccination [6–8]. Standard serological methods like virus neutralization test (VNT), agar gel immunodiffusion, indirect fluorescence antibody test (IFAT) and Western blot are described [9, 10].

These tests are expensive and time consuming therefore limiting their use for fast serological screening of cattle populations. The only test validated to date by the OIE is the VNT, coming along with high specificity and good sensitivity, but reduced high-throughput application. As performing VNT includes the handling of live capripoxvirus, its application can face additional restrictions [11]. The IFAT comes along with the disadvantage of increased cross reactivity with bovine papular stomatitis virus and other poxvirus antibodies.

For efficient immunological investigations at large scale, Enzyme-linked Immunosorbent Assay (ELISA) has been found to be more suitable compared to the above-mentioned serological methods. Few studies on the development of ELISA tests for LSD-specific antibody detection by using recombinant P32 [12], recombinant two virion core protein of sheeppox virus [13], and inactivated sheeppox virus as coating antigen for antibody detection [14] have been published. A double antigen ELISA from the IDvet® is currently the only commercially available kit for detection of capripox specific antibodies applied in field studies for seromonitoring [15]. All of these ELISA have been developed for plasma or serum as sample matrix to be used.

Apart from blood, the detection of specific antibodies is also possible from other matrices such as milk. Cow milk contains three major classes of immunoglobulin’s (Ig): IgG, IgM and IgA [16]. Dominant class of immunoglobulins in milk, comprising about 65% of the total, is IgG. The concentration of IgG varies between serum and mammary secretions. Highest concentrations can be found in colostrum (32–212 mg/ml), followed by serum (25.0 mg/ml) and finally in milk with about 0.72 mg/ml [17]. As for the colostrum, a steady gradual decrease of antibody levels is described each hour [18]. The concentration of antibodies in regular milk is much lower than that in colostrum and depends on factors like clinical or subclinical mastitis, age, breed, feeding system and the stage of lactation. In addition, primiparous cows are described to have colostrum and milk with overall lower levels of immunoglobulins compared to multiparous cows [18, 19].

Using milk for the detection of antibodies by ELISA has been found suitable targeting antibodies against viruses such as bovine viral diarrhea virus (BVDV) [20], bluetongue virus [21, 22] and bovine alpha herpesvirus 1 (BHV-1) [23]. As milk samples are non-invasive and cheaper to collect compared to serum and plasma, this method has been widely used in mass screening activities targeting either individual animals or bulk milk samples for the determination of the immunological status at animal or herd level respectively.

In this study, we investigated the suitability of milk, as individual animal and bulk sample to detect LSD-specific antibodies using a commercially available capripox ELISA designed for serum and plasma samples.

## Material and methods

### Sample material

For the purpose of this study, serum and milk samples were collected from 154 lactating dairy cows from farms in the Kraljevo municipality in Serbia. This area had no evidence of previous LSD field virus circulation. All cows included in this study were vaccinated in 2016 with a Neethling vaccine strain (Onderstepoort Biological Products, South Africa) and re-vaccinated in 2017 with BOVIVAX LSD-N Neethling vaccine (M.C.I. Sante Animale, Morocco). During sampling, all animals were clinically examined. Animals showing any generic clinical signs of disease were excluded from study. Blood samples were collected using blood colleting tubes without anticoagulant BD Vacutainer^tm^4055269 (Belliver Industrial Estate, UK) via coccygeal venipuncture. Extracting serum from blood was done by centrifugation at 2000 RPM for 20 min. Milk samples were collected in 15 ml sterile plastic tubes, individual milk samples by hand milking from all teats and bulk milk from milk tank of the farm. Milk samples were conserved using 1% sodium-azide. All obtained samples were aliquoted in 1.5 ml centrifugal tubes and stored at − 20 °C until further examination. Repository samples of cattle from Germany (*n* = 352) were included in this study as negative control panel since Germany is LSD-free and the vaccination against LSDV is forbidden.

### Elisa

For the detection of LSD-specific antibodies in serum and milk samples a commercial ELISA test for serum and plasma (ID Screen® Capripox Double Antigen Multi-species ELISA, IDvet, Montpellier, France) was used. Investigation of serum and milk (individual and bulk) samples was performed according to manufacture protocol for serum and plasma. Additionally, investigation of milk (individual and bulk) samples was performed with modifications of sample incubation time, from 90 min at + 21 °C to overnight incubation at + 4 °C. All milk samples were centrifuged at 10000 rpm for 10 min; fat supernatant was removed and only the liquid fraction was used for analysis.

### Statistical analysis

Statistical analysis was performed using RStudio© Version 1.1.456 (Boston, USA) with the packages Optimal Cutpoints Version 1.1–3 and reshape2 1.4.3 from the CRAN repository. Receiver operating characteristic (ROC) analysis was performed for determination of the Youden index and determination of the most suitable cut-off value for maximum specificity for milk samples using serum ELISA results with as reference. The McNemar test was performed to determine the statistical significance between results of the two ELISA protocols used for the milk samples.

## Results

### Sampling and clinical examination

No clinical mastitis or any generic clinical signs of disease were observed during sample collection. During the 2017 sampling period, no new LSD outbreak was reported in Serbia. In total 154 individual and 38 bulk milk samples were collected. The 38 obtained bulk milk samples comprised of milk from two to ten individuals depending on the overall farm size and the number of animals in lactation.

### Antibody detection

**Fig. 1:**
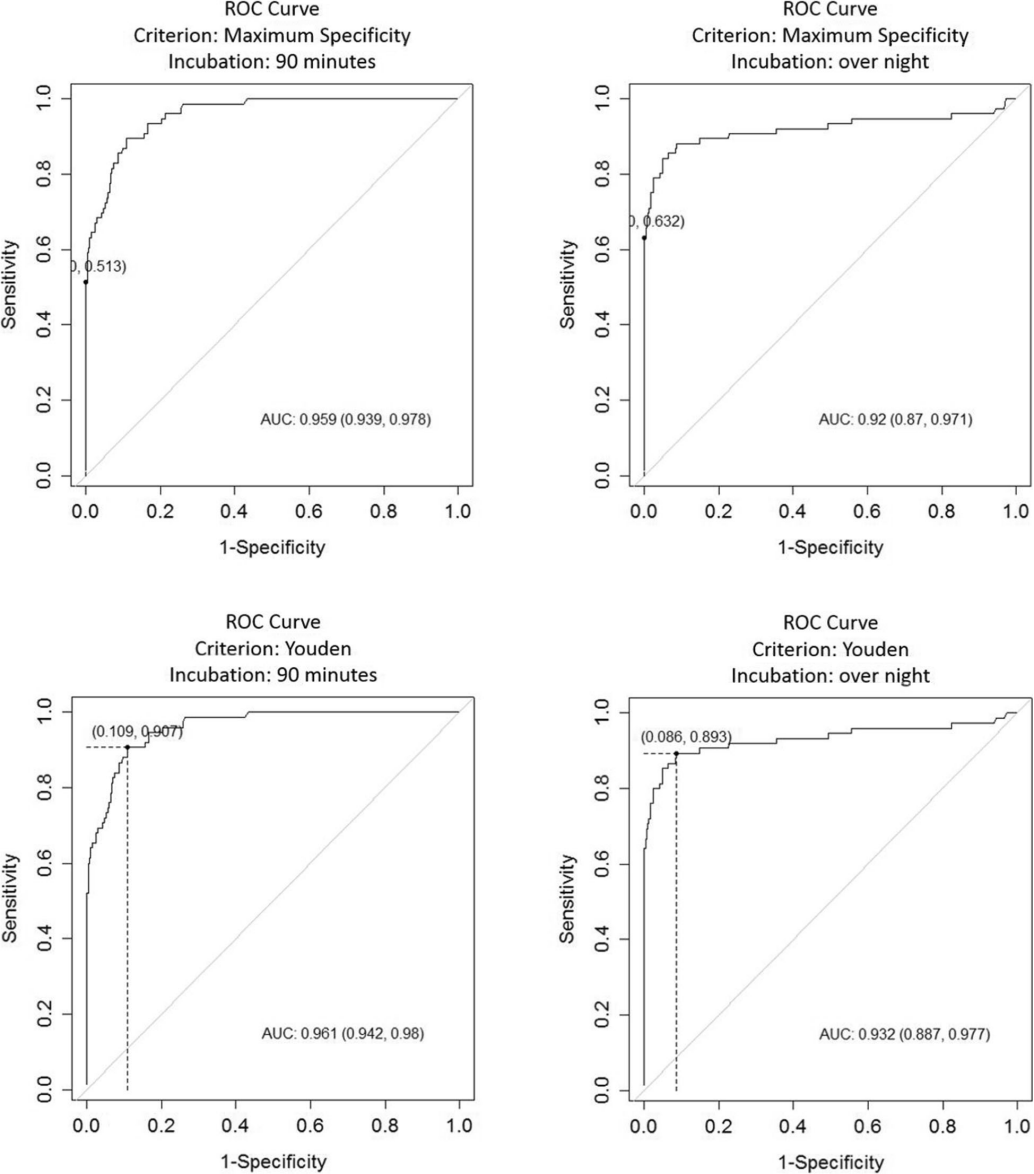
Comparative view on ROC curves of milk samples analyzed by ELISA with different criteria and incubation times

From 154 investigated serum samples, 75 were detected as positive with ELISA test. The results obtained from the serum served as reference for the ROC analysis evaluating the two different protocols used on the milk samples to determine the respective cut-off values for the criteria Youden and maximum specificity. The results are summarized in Table 1 and Fig. 1.

**Table 1:** Comparative ROC analysis of milk samples with 90 min and overnight incubation time using Youden and maximum specificity as criteria

Criterion	Youden		Maximum specificity	
Incubation	90 min	overnight	90 min	overnight
Area under ROC curve (ACU)	0.961 (0.942, 0.98)	0.932 (0.887, 0.977)	0.959 (0.939, 0.978)	0.92 (0.87, 0.971)
cut-off	1.308	3.086	9.425	9.785
Se	0.895	0.882	0.513	0.632
Sp	0.891	0.914	1.0	1.0
PPV	0.591	0.644	1.0	1.0
NPV	0.980	0.978	0.921	0.939
DLR. Positive	8.186	10.222	Inf	Inf
DLR. Negative	0.118	0.13	0.487	0.368
FP	47.0	37.0	0.0	0.0
FN	8.0	9.0	37.0	28.0
Optimal criterion	0.785	0.795

Following these cut-off determinations, all further analysis of the milk samples used the cut-off of ≥10 as determined through the maximum specificity criterion.

**Fig. 2:**
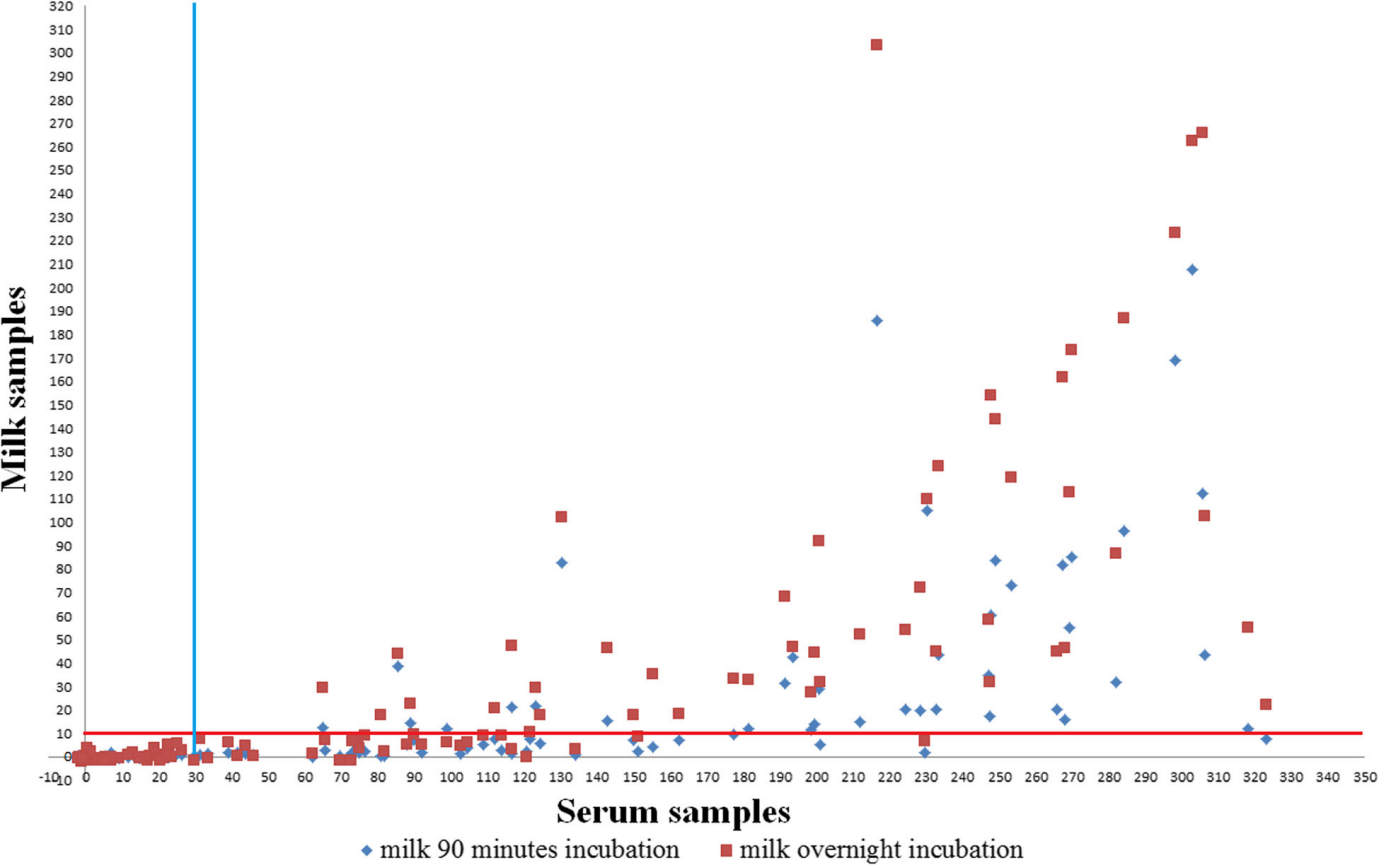
Detected S/P% values of serum samples and their respective milk samples following two incubation protocols. Cut-off for serum at 30 as provided by manufacturer and for milk set at 10 according to ROC analysis criterion maximum specificity

From the tested 154 milk samples, samples from 38 animals tested positive when applying the proposed manufacturer protocol for serum and plasma with a 90 min incubation time. The number of positive samples from the same sample set increased to 48 when using the modified overnight incubation without creating false positive results in reference to the serum. The relationship of the results on milk samples using two different protocols, one treating milk samples identical to the serum samples and the other using an overnight incubation time is depicted in Fig. 2. The results between the two incubation protocols are significantly different (*p* < 0.05), with overnight incubation obtaining higher S/P% values.

The overall agreement between investigated serum and milk samples with both protocols is shown in Table 2, stratified additionally by the level of positivity in the serum ELISA. Higher levels of agreement are achieved the higher the serum ELISA values are.

**Table 2:** Comparison of detected positive and negative milk samples to detected S/P% values of positive serum samples

Positive serum samples		Individual milk samples			
		90 min incubation		Overnight incubation	
S/P% value	Number	Positive	Negative	Positive	Negative
≥201	28	25 (89.29%)	3 (10.71%)	27 (96.43%)	1 (3.57%)
101–200	24	9 (37.50%)	15 (62.50%)	16 (66.67%)	8 (33.33%)
30–100	23	4 (17.39%)	19 (82.61%)	5 (21.74%)	18 (78.26%)

**Fig. 3:**
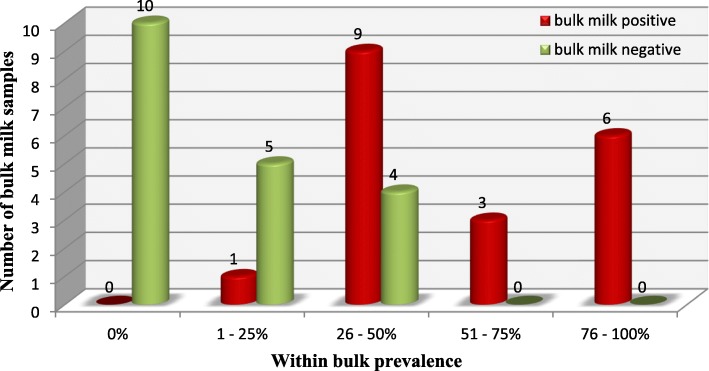
Bulk milk results stratified over the within bulk prevalence determined through individual milk testing; overnight incubation and cut-off value ≥10

The sensitivity of ELISA for the bulk milk sample is however reduced over the testing of the individual animal whilst the specificity remained high. No false positive bulk milk samples appeared in relation to obtained results of individual milk samples composing it. Bulk prevalence had a close relation with bulk milk results showing better performance of bulk milk samples, which had higher prevalence. The composition and results of 38 bulk milk samples are summarized in Fig. 3, for details of the bulk milk composition refer to Additional file 1.

Except two samples from Germany, which had borderline result using overnight incubation, the rest of the samples reacted negative.

## Discussion

In the light of the LSD expansion in recent years, tools to determine the immune status of animals and populations are on demand to facilitate control and eradication efforts. The obtained results from this study provide the proof of principle, that milk can be used as sample for the detection of antibodies against LSDV both at the individual animal and herd level.

Applying overnight incubation for milk samples at + 4 °C in ELISA tests has been described as suitable for the improved detection of specific antibodies for different viruses [20, 23, 24]. In this study, where no further modifications on neither the milk sample nor the ELISA kit have been applied, the increased incubation time confirms to be suitable to achieve higher test sensitivity.

The determination of the optimal cut-offs for will depend on the actual purpose of the testing. In this study higher cut-off value aiming for maximum specificity was taken for further analysis, accommodating a situation where false positive samples would have a higher negative impact than false negative, as all animals were exposed to LSDV through vaccination. For a final confirmation, serum samples could be collected and analyzed by ELISA or other serological tests recommended by OIE [1] if needed.

In general, observed mismatches of detected positive serum and negative milk samples together with lower S/P% values in milk samples, could be attributed to lower concentration of immunoglobulins in milk which is multifactorial [18, 19]. Improvement of the ELISA test performance for the detection of LSD-specific antibodies in milk samples can probably be addressed by purification of immunoglobulins using commercially available kits or by precipitation of proteins using ammonium sulfate [25]. On the other side, Klintevall et al. [24] showed in their study that cows that suffered of clinical mastitis may give false positive ELISA results when investigating milk. Cows included in this study did not suffer from clinical mastitis and therefore this aspect was not observed.

The results obtained in this study were not put in relation to the exact date of vaccination of the animals. It is well described however that the levels of detectable antibodies in the serum vary and will increase between one and 12 weeks post vaccination, after which period antibody titer will slowly start to decrease [6]. Similar developments of titers are likely to be seen in the milk especially after vaccination campaigns stop. In case of a LSDV re-emergence, the detection of positive milk samples will imply that animals were in contact with the virus provoking antibody synthesis.

One additional step forward in exploring the diagnostic advantages of milk samples was the detection of LSD-specific antibodies in pooled milk samples representing milk bulks typical for Serbia. Benefits of using bulk milk samples have been described previously for fast preliminary immunological investigation at herd level in response of determination of seroprevalence against BVDV and BHV-1 [26]. The detection of antibodies from bulk milk mainly depends of the number of positive animals included in bulk milk and the concentration of immunoglobulins in positive milk included in the bulk milk. Even though the number of animals per bulk in this study was not too big, detection of positive bulk milk samples was possible starting with bulk prevalence of 25%. As expected, higher bulk milk prevalence delivered better results by detecting all positive bulk milk samples which had prevalence higher that 50%. According to obtained bulk milk results and described LSD morbidity rate of 45% [27], determination of LSDV seroprevalence using bulk milk samples can be suitable. Multiple testing of bulk milk can be additionally recommended on a wider scale for screening purposes which will provide robust, but fast herd level results. In addition, these results should be validated for settings with larger animal numbers contributing to the bulk milk.

## Conclusion

The commercially available ELISA kit from IDvet for the detection of capripoxvirus specific antibodies is in principle suitable to be used on milk samples, from individual animals as well as pooled milk samples of small bulks. Cut-off values will need to be specified according to the purpose of testing. Additional modifications either on the ELISA kit or through antibody enrichment steps can lead to improve the sensitivity and specificity and will make this approach a time and cost efficient, non-invasive monitoring for LSD occurrence or LSD vaccination in the field.

## Supplementary information

**Additional file 1.** Table providing an overview on bulk milk sample composition and the respective ELISA results.

## Data Availability

The datasets used and/or analysed during the current study are available from the corresponding author and/or the first author on reasonable request.

## Abbreviations

BHV-1: Bovine alpha herpesvirus 1
BVDV: Bovine viral diarrhea virus
ELISA: Enzyme-linked Immunosorbent Assay
IFAT: Immunofluorescent antibody test
Ig: Immunoglobulin
LSD: Lumpy skin disease
LSDV: Lumpy skin disease virus
npv: Predictive negative value
ppv: Predictive positive value
ROC: Receiver operating characteristic
S/P %: Percentage of positivity compare to the positive control
Se: Sensitivity
Sp: Specificity
VNT: Virus neutralization test
