# Supplementary material for: Suitability of individual and bulk milk samples to investigate the humoral immune response to lumpy skin disease vaccination by ELISA
Source: Virol J. 2020 Mar 5;17:28. doi: 10.1186/s12985-020-01298-x (PMC7059690; doi:10.1186/s12985-020-01298-x)
Supplement: Supplementary file 1 — Additional file 1. Table providing an overview on bulk milk sample composition and the respective ELISA results. [file 12985_2020_1298_MOESM1_ESM.docx]

**Additional file 1:** Table providing an overview on bulk milk sample composition and the respective ELISA results.

| **Number of animals in bulk** | **Number of positive animals in bulk** | **Within Bulk prevalence** | **Bulk milk result** |
| --- | --- | --- | --- |
| 3 | 1 | 33% | Pos |
| 4 | 0 | 0% | Neg |
| 4 | 0 | 0% | Neg |
| 2 | 1 | 50% | Neg |
| 2 | 1 | 50% | Pos |
| 3 | 1 | 33% | Neg |
| 2 | 0 | 0% | Neg |
| 3 | 0 | 0% | Neg |
| 7 | 0 | 0% | Neg |
| 10 | 1 | 10% | Neg |
| 6 | 0 | 0% | Neg |
| 6 | 0 | 0% | Neg |
| 2 | 2 | 100% | Pos |
| 4 | 1 | 25% | Neg |
| 4 | 3 | 75% | Pos |
| 2 | 1 | 50% | Pos |
| 3 | 1 | 33% | Pos |
| 2 | 0 | 0% | Neg |
| 3 | 3 | 100% | Pos |
| 7 | 4 | 57% | Pos |
| 10 | 2 | 20% | Neg |
| 6 | 2 | 33% | Neg |
| 6 | 2 | 33% | Pos |
| 3 | 3 | 100% | Pos |
| 2 | 1 | 50% | Pos |
| 4 | 0 | 0% | Neg |
| 3 | 2 | 66% | Pos |
| 2 | 2 | 100% | Pos |
| 2 | 2 | 100% | Pos |
| 2 | 2 | 100% | Pos |
| 2 | 1 | 50% | Pos |
| 2 | 1 | 50% | Pos |
| 4 | 0 | 0% | Neg |
| 2 | 1 | 50% | Neg |
| 5 | 2 | 40% | Pos |
| 9 | 2 | 22% | Neg |
| 5 | 1 | 20% | Neg |
| 4 | 1 | 25% | Pos |
